# Stomach Movements before and after Gastro-Jejunostomy

**Published:** 1909-01-09

**Authors:** 


### STOMACH MOVEMENTS BEFORE AND AFTER GASTRO-JEJUNOSTOMY.

Investigations upon the normal and abnormal movements of the alimentary canal have been made by several observers in this country since the method of applying the *x*-rays after the oral administration of bismuth soup has been introduced. There is an excellent paper upon the subject by Dr. A. F. Hertz in the "Guy's Hospital Reports," vol. lxi.; another by Mr. H. M. W. Gray in the *Lancet* for July 28, 1908; a third by Dr. Morton in the *British Medical Journal*; and so on. Many time-honoured assumptions in regard to the movements of the intestines have been confirmed; some others have been shown to be erroneous. It is of the stomach movements especially that the latter has been the case.

By the use of the purest carbonate of bismuth no ill-effects arise, even when amounts large enough to give a shadow of the entire stomach are swallowed. Poisonous symptoms recorded by some observers may have been due to impurities in the samples of bismuth employed.

It would seem that the stomach, from a motor as well as from a secretory point of view, consists of two entirely separate portions separated by a special muscle band to which Mr. Gray applies the term "middle sphincter." The normal living stomach often presents an hour-glass appearance when examined under the *x*-rays by the bismuth method, owing to the contraction of this middle sphincter. A precisely similar constriction is often seen in stomachs that are examined post-mortem, disappearing as soon as the middle sphincter is cut through. It seems that the churning movements which are usually attributed to the whole stomach are really confined to the cardiac portion, proximal to the middle sphincter; apparently the movements of the pyloric tube—that is to say, the portion of stomach between the middle sphincter and the pylorus—are not of a churning, but of a true peristaltic nature; and that whereas food remains on the proximal side of the middle sphincter for as much as three or four hours, it remains but a short time in the pyloric tube when once it has passed the middle sphincter. Cardiac and pyloric portions act in great part independently of one another.

Mr. Gray has made a study of the conditions that hold good as regards the motor functions of the stomach after gastro-jejunostomy. He finds that in cases where the muscular power of the stomach is still very fair the cardiac portion of the viscus does not empty more quickly after "correct" gastro-jejunostomy than it did before it, some food still remaining on the proximal side of the middle sphincter four hours after a light meal. The pyloric portion, on the other hand, empties much more quickly than it was able to before. In a typical case the black bismuth shadow could be seen passing through the stoma within five minutes of its entry into the pyloric tube.

One very practical outcome of these investigations would seem to be in connection with the best site for a gastro-jejunostomy opening. Food does not appear to be ready for leaving the stomach as long as it is on the proximal side of the middle sphincter. If, therefore, there is no good reason to the contrary in any particular case, it is clearly much wiser to make sure that the artificial stoma is in the pyloric tube, and not in the cardiac sacculus of the stomach. If the stomach acts uniformly throughout as regards its motor functions, it would, one might think, matter little whether the gastro-jejunostomy opening is at the pylorus or nearer the fundus, so long as the lowest portion of the viscus is opened into. This is not the case. The physiology of the motor functions of the stomach indicates a clear necessity for making the gastro-jejunostomy opening upon the distal side of the middle sphincter wherever possible.

One other practical result follows, and that is in regard to the after-treatment of cases of gastro-jejunostomy for extreme gastrectasis. The great dilatation of the stomach in these cases has caused the "middle sphincter" to be so over-stretched that there is no longer any line of demarcation between the cardiac sacculus and the pyloric canal. The whole viscus has become one huge dilated bag. The mere performance of gastro-jejunostomy in such a case will not immediately restore the middle sphincter to its proper contractility. The patient will feel so much better than before that he will be tempted to eat abundantly in sheer enjoyment; the food will not be prevented from getting at once into the pyloric canal, where it should not be present until it has been fully dealt with in the cardiac end of the stomach. Recurrence of gastric symptoms occurs almost at once to the disappointment both of patient and of doctor. The point to bear in mind is that, before the patient can expect to eat with impunity in such a case, time must be given to the over-stretched middle sphincter to recover its tone and to restore the stomach to its normal condition of a viscus in two compartments.

Mr. Gray's advice may best be given in his own words: "Such observations reasonably indicate the following post-operative treatment in cases of excessive dilatation in order to obtain the greatest good from such gastric juices as may still be secreted. (1) Prevent gorging. In the absence of the middle sphincter and sufficient cardiac peristalsis there cannot be efficient mixing of the food with the acid cardiac juices. The larger the quantity of food ingested the less satisfactory will the mixing be. (2) Recommend rest after meals, especially after the chief meal of the day. The supine position should be assumed for half an hour at least so that the food is prevented from gravitating toward the pyloric stoma and being prematurely evacuated from the stomach. (3) Caution against eating just before retiring to bed, as after the patient lies down food may remain overnight in the flaccid atonic cardiac portion and give rise to nausea, heaviness, headache, etc., in the morning. These directions will insure the best results, and from time to time some such regulations are necessary. The unspeakable relief afforded by gastro-enterostomy to patients with dilated stomachs sometimes tempts them to excess in eating and drinking for which they suffer.

